# Factors affecting the number of sentinel lymph nodes removed in patients having surgery for breast cancer

**DOI:** 10.1007/s10549-020-05843-8

**Published:** 2020-08-18

**Authors:** J. Michael Dixon, Julia Grewar, Dominique Twelves, Ashley Graham, Carlos Martinez-Perez, Arran Turnbull

**Affiliations:** 1 Edinburgh Breast Unit and Breast Cancer Now Group, MRC Institute of Genetics and Molecular Medicine, University of Edinburgh, Edinburgh, UK; 2 Edinburgh Breast Unit, NHS Lothian, Edinburgh, UK; 3 Medical School, University of Edinburgh, Edinburgh, UK; 4 Department of Pathology, NHS Lothian, Edinburgh, UK

**Keywords:** Sentinel lymph node biopsy, Breast cancer, Treatment, Staging, Axilla

## Abstract

**Purpose:**

The goal of sentinel lymph node biopsy is to establish the presence or absence of cancer cells in regional axillary nodes. The number of sentinel nodes harvested from each patient varies. The aim of this study was to determine what factors influence the number of sentinel nodes excised at sentinel node biopsy.

**Methods:**

Data from 426 patients with breast cancer who underwent sentinel lymph node biopsy at the Edinburgh Breast Unit by 10 different experienced breast surgeons were included in this analysis. Univariate and multivariable statistical analysis was performed.

**Results:**

In the multivariate analysis the number of sentinel nodes biopsied varied significantly between operating surgeon (*p* < 0.0001) and was also statistically associated with the use of neoadjuvant chemotherapy (*p* < 0.0001) and with the number of involved lymph nodes (*p* < 0.0001). More nodes were removed in patients who received neoadjuvant chemotherapy and had metastases in sentinel lymph nodes.

**Conclusions:**

This study shows that the surgeon plays a pivotal and significant role in determining the numbers of sentinel nodes removed by sentinel lymph node biopsy. Surgeons should monitor their own data on the average numbers of sentinel nodes they remove. Some surgeons may not be removing sufficient numbers of sentinel nodes to maintain a low false negative rate for this procedure.

**Electronic supplementary material:**

The online version of this article (10.1007/s10549-020-05843-8) contains supplementary material, which is available to authorized users.

## Introduction

The lymphatic system was discovered in 1653 by Bartholin. Virchow in the nineteenth century believed that lymph nodes filtered lymph [1]. The spread of cancer cells through the lymphatic system by a defined route stemmed from these observations and resulted in the technique of sentinel lymph node biopsy (SLNB). A sentinel node is named after the first guard who stands watch. Lymph flow from the tumour site passes through the first nodes draining the breast known as sentinel nodes [1]. A negative sentinel node biopsy assumes no metastasis to any axillary nodes. Metastatic breast cancer is present when cancer cells involve the sentinel node or nodes [2]. SLNB provides prognostic information and guides adjuvant treatment.

SLNB utilises intra-operative mapping of breast lymphatic drainage usually with a blue dye and isotope [1]. A sentinel lymph node in breast cancer surgery is defined as a blue node or a “hot” radioactive node and includes nodes other than the hottest node providing that it contains at least 10% of the radioactivity of the hottest node [3]. The false negative rate of SLNB in breast cancer surgery falls with an increase in sentinel nodes removed [4]. In the NSABP B-32 trial [5] one node gave a false negative rate of 17.7% whereas three or more nodes had a false negative rate of 4.8%. There remains much variation between how many nodes surgeons remove and what comprises an adequate SLNB.

The primary aim of this study was to identify what factors influence the number of lymph nodes removed by sentinel lymph node biopsy. This was assessed in the context of patients undergoing surgical treatment and sentinel node biopsy for breast cancer at the Edinburgh Breast Unit.

## Methods

### Database and study population

Data were collected retrospectively from the Edinburgh Breast Unit at the Western General Hospital, Edinburgh with the aim of acquiring 50 patients for each of the 10 experienced breast surgeons. Patients with core biopsy diagnosed invasive or in situ breast cancer who underwent SLNB as part of breast conserving surgery or mastectomy were eligible for inclusion in this study. Surgery dates ranged from March 2016 to September 2017. The hospital record system, pathology reports and operating theatre diaries were used to identify patients. Sentinel node were identified using 25 MBq of Technetium injected by the surgeon immediately after the induction of anaesthesia either under the nipple or intradermally over the cancer combined with 2 ml of Patent blue V injected in the sub and periareolar regions [6]. The breast was then massaged. No scintiscans were performed. The technique was standardised and all surgeons were trained in the technique by JMD as part of his requirements as a radioisotope license holder [6]. The surgeons included in this study were consultants, associate specialists or staff grade pure breast surgeons who had been performing sentinel node biopsy for at least 5 years. Only axillary sentinel nodes were sampled. The standard definition of sentinel nodes was used in this study, that is nodes that were blue or had isotope counts of at least 10% of the hottest node. The number of sentinel nodes removed and used for analysis was the number found and reported by the pathologist. Surgeons also removed other axillary nodes if they were palpable or felt abnormal and these were recorded as sampled nodes.

### Population characteristics

Four hundred and forty‐three patients had a histologically confirmed diagnosis of invasive or in situ breast cancer and had SLNB performed in the Edinburgh Breast Unit by one of ten surgeons. Seventeen patients were excluded because there was incomplete data on sentinel node numbers or SLNB was not performed. In patients who had a complete pathological response to neoadjuvant therapy, tumour size was not included in the analysis. An oestrogen receptor (ER) positive cancer was defined as a cancer with > 1% cells staining positively for ER by immunohistochemistry. ER levels were classified on the Allred scale ranging from 0 to 8.

### Statistical analysis

Distribution of data was determined to be non-normal using the Kolmogorov–Smirnov test and testing for equivalent mean and variance. Univariate analysis was performed using appropriate statistical tests as outlined in the results section. Multivariable analysis was carried out using IBM SPSS software to build a Poisson log linear main effects generalised linear model analysis based on the Wald Chi-square statistic. Data collected included patient age, tumour size, tumour grade, type of surgery and surgeon, neoadjuvant chemotherapy (NAC), lympho-vascular invasion, number of confirmed positive sentinel lymph nodes and hormone and HER2 receptor status. Separate subset analyses were performed including and omitting patients who either (i) received neoadjuvant chemotherapy or (ii) had an involved node or nodes at diagnosis and received neoadjuvant chemotherapy, all of whom had one involved node clipped at diagnosis. *p* values ≤ 0.05 were considered to be of significance.

## Results

### Patient characteristics

Table 1 displays patient cohort data for a cohort of 426 patients who met the inclusion criteria in relation to each variable for which data were collected. Average patient age was 61 years. The mean tumour size of this cohort was 22.7 mm and the most commonly confirmed grade was grade 2 disease.

**Table 1:** Baseline characteristics of patient cohort displayed in relation to each variable

Variable	Number	% of cohort (*N* = 426)
Number of sentinel nodes biopsied		
0	17	4.0
1–2	251	58.9
≥ 3	158	37.1
Total number of nodes removed including 426 sentinel nodes		
0	0	0
1–2	287	53.4
≥ 3	250	46.6
Histologically confirmed node status		
Node negative	362	85.0
Node positive	64	15
Histologically confirmed positive nodes by number of sentinel nodes biopsied		
Node positive with 1–2 sentinel nodes biopsied	27/251 (10.7%)	6.3
Node positive with ≥ 3 sentinel nodes biopsied	33/158 (20.8%)	7.7
Patient age (years) at surgery		
30–40	14	3.3
41–50	58	13.6
51–60	119	27.9
61–70	148	34.7
71–80	64	15.0
81–90	16	3.8
91–100	3	0.7
Missing data	4	0.9
Neoadjuvant chemotherapy		
Yes	40	9.4
No	386	90.6
Lymphatic/vascular invasion		
Yes	61	14.3
No	364	85.4
Indeterminate	1	0.2
Primary type of surgery		
WLE SLNB	365	85.7
MX SLNB	36	8.5
Re-ex SLNB	10	2.3
SLNB alone	15	3.5
Grade		
1	104	24.4
2	233	54.7
3	71	16.7
Unknown	18	4.2
Tumour size (mm)		
< 10	61	14.3
10–20	190	44.6
> 20	157	36.8
Unknown	18	4.2
Tumour hormone status		
ER + HER2 +	16	3.8
ER + HER−	321	75.4
ER−HER2 +	24	5.6
ER−HER2−	54	12.7
Unknown	11	2.6

*SLNB *sentinel lymph node biopsy,* WLE *wide local excision,* MX *mastectomy,* RE-EX* re-excision,* DCIS/LCIS *ductal/lobular carcinoma in situ,* ER *oestrogen receptor*, HER2 *human epidermal growth factor receptor 2

Sentinel nodes were identified in 96.1% of patients in the cohort. In 17 patients, (3.9% of the cohort,) no sentinel nodes were identified. These patients had an axillary node sampling procedure and had an average of 4.5 axillary lymph nodes removed. This procedure has been validated in Edinburgh by a series of randomised trials. Only two of the patients (11.7%) having axillary sampling had histologically confirmed involved axillary lymph nodes.

**Fig. 1:**
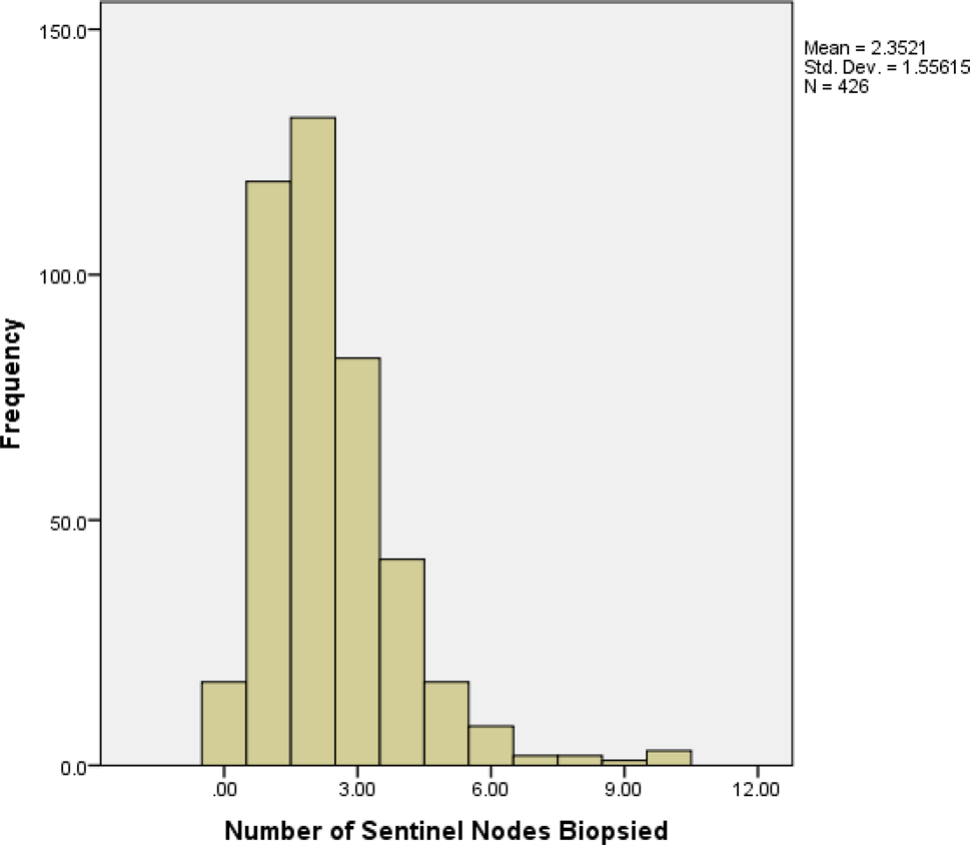
Histogram of number of sentinel nodes removed per SLNB procedure (frequency). Total number procedures, *n* = 444. See Online Appendix for raw data

The number of nodes removed per SLNB procedure across all patients (*n* = 426) is detailed in Fig. 1. The mean number of nodes removed was 2.35. As shown in Fig. 1, the data is non-normally distributed.

### Univariate analysis

Univariate analysis revealed statistically significant associations between the number of sentinel lymph nodes sampled and surgeon (*p* < 0.0001), age of the patient (*p* < 0.0001), the use of neoadjuvant chemotherapy (*p* < 0.0001), number of confirmed histologically proven involved (positive) sentinel lymph nodes (*p* < 0.0001), tumour size (*p* < 0.001), tumour grade (*p* > 0.003) and ER status (*p* < 0.008). Greater numbers of sentinel lymph nodes were removed in women who were younger, received neoadjuvant chemotherapy, had larger tumours, had more involved axillary nodes, had higher grade cancers and had low ER levels (Allred scores < 5) or were ER negative.

### Surgeon

**Fig. 2:**
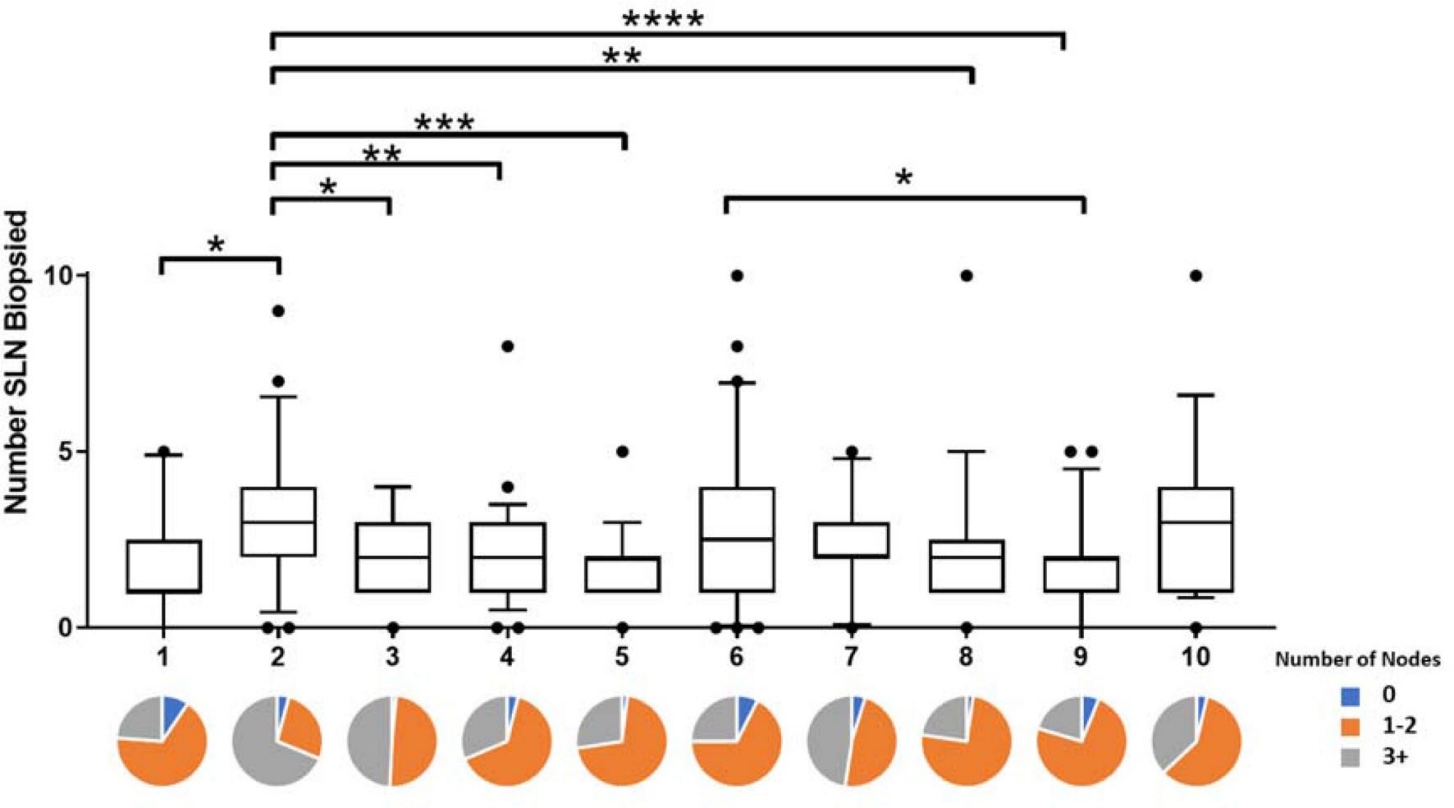
Boxplot graph showing the mean, upper and lower quartiles and 95% confidence intervals for number of SLNs biopsies for each surgeon (1–10). Stars represent statistical significance: * < 0.05, ** < 0.01, *** < 0.001, **** < 0.0001). Pie charts under the graph illustrated the proportion of patients for each surgeon where no (blue), 1–2 (orange) or > 3 (grey) sentinel nodes were biopsied

Ten surgeons performed SNB with number of procedures per consultant ranging from 21 to 78 with a mean of 45.7 (Fig. 2).

Univariate analysis using the Kruskal–Wallis test with Dunn’s multiple comparisons test showed a significant association between surgeon and number of sentinel lymph nodes biopsied (*p* < 0.0001) (Fig. 2). In particular, surgeon 1 took a statistically significantly higher number of sentinel lymph nodes than surgeons 1, 3, 4, 5, 8 and 9. Nine of the ten surgeons took an average of less than three nodes per procedure.

### Number of confirmed positive sentinel lymph nodes

**Fig. 3:**
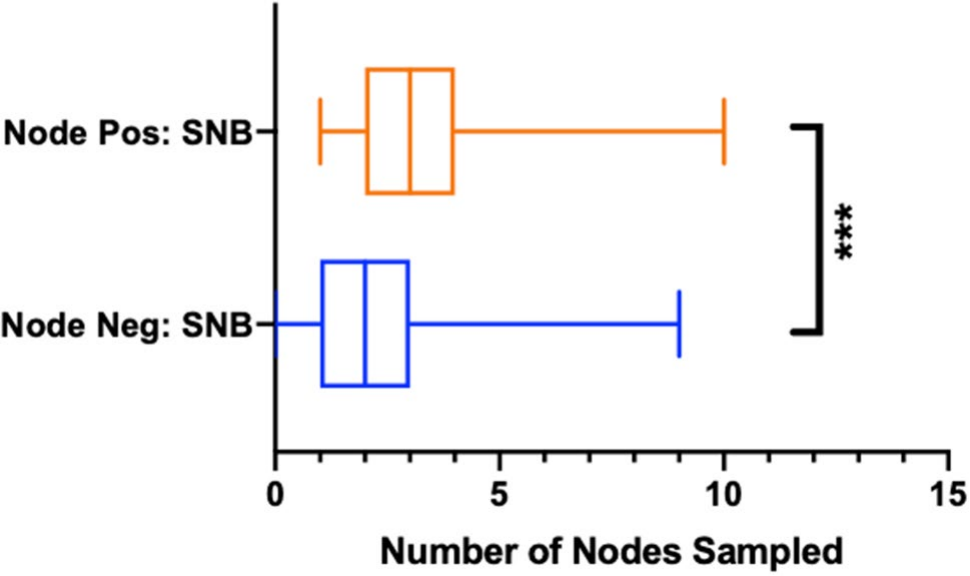
Boxplot graph showing the mean (2.23 for node negative and 3.03 for node positive), upper and lower quartiles and 95% confidence intervals comparing sentinel nodes sampled between patients who were histologically determined to be node negative and those who were node positive. Blue: node negative, orange: node positive. Stars represent statistical significance: *** < 0.001)

Univariate analysis performed using the Mann–Whitney test revealed that significantly (*p* = 0.0028) greater numbers of sentinel lymph nodes were taken from patients who were subsequently confirmed to be node positive compared to the numbers of sentinel node samples from patients with node negative disease (Fig. 3).

### Neoadjuvant chemotherapy (NAC)

**Fig. 4:**
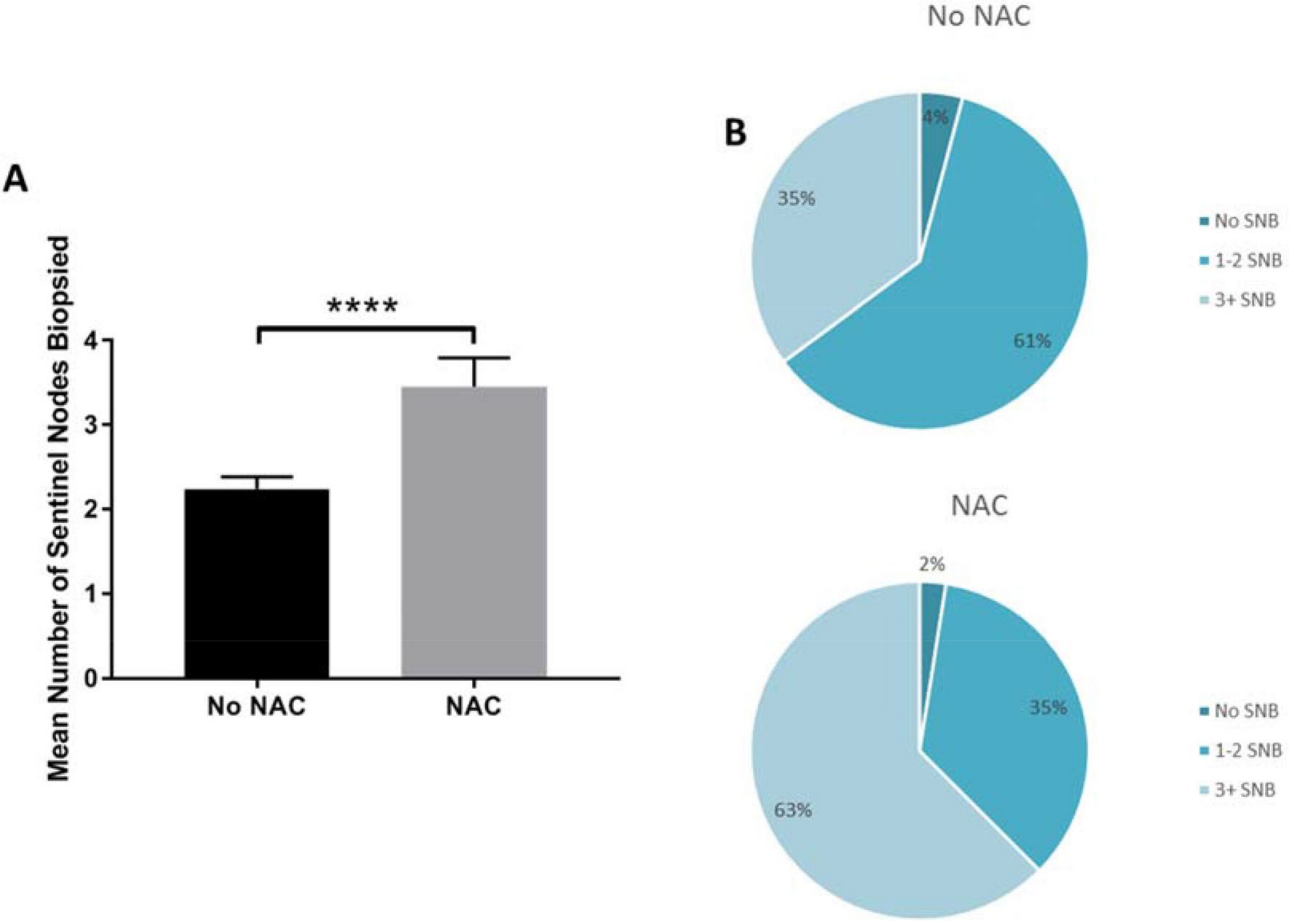
**a** Comparison of mean with 95% confidence intervals of number of SLN’s removed at biopsy in patients who did and did not receive NAC. Stars denote *p*-value < 0.0001 using Mann–Whitney statistics. **b** Pie charts of percentages of patients who had no, 1–3 or 4  + sentinel node biopsies split by those who did (lower) and did not (upper) receive NAC

The average number of sentinel nodes excised in relation to whether patients had NAC is displayed in Fig. 4. Data regarding NAC were available for 426 patients, of whom 40 (9.38%) received NAC. Those who received NAC in this study had a mean of 3.5 nodes sentinel lymph nodes removed, whereas those without NAC had a mean of 2.2 nodes, statistically significant using the Mann–Whitney test (3.5 ± 2.2 vs 2.2 ± 1.4, *p* < 0.0001). Of the cohort who received NAC (*N* = 40), 62.5% had 3 or more sentinel lymph nodes removed compared to 37% of the cohort who did not receive NAC. When sampled nodes are included, the mean number of nodes removed increased to 4.6 and 83.3% of patients after NAC had 3 or more nodes sent to pathology.

Twenty-eight of the 40 patients (70%) who received NAC were node negative at diagnosis, the mean number of sentinel nodes removed in this group was 3.32 and 18 of these 28 patients (64.2%) had 3 or more sentinel nodes removed. In the 10 patients who had less than 3 sentinel nodes removed 5 (50%) had other nodes sampled increasing the mean number of nodes removed in these 10 patients from 1.5 to 2.8.

Twelve women who received NAC were node positive at diagnosis and the mean number of sentinel nodes removed in these patients was 3.75. Seven of these 12 patients (58.3%) had 3 or more sentinel nodes removed but when sampled nodes were included, 10/12 (83.3%) had 3 or more nodes sent to pathology.

### Generalised linear modelling for multivariable analysis

Multivariable analysis in the whole series showed a significant association between the number of nodes removed at sentinel node biopsy and surgeon (*p* < 0.0001), the use of neoadjuvant chemotherapy (*p* < 0.0001) and number of involved sentinel lymph nodes (*p* < 0.0001) (Table 2).

**Table 2:**
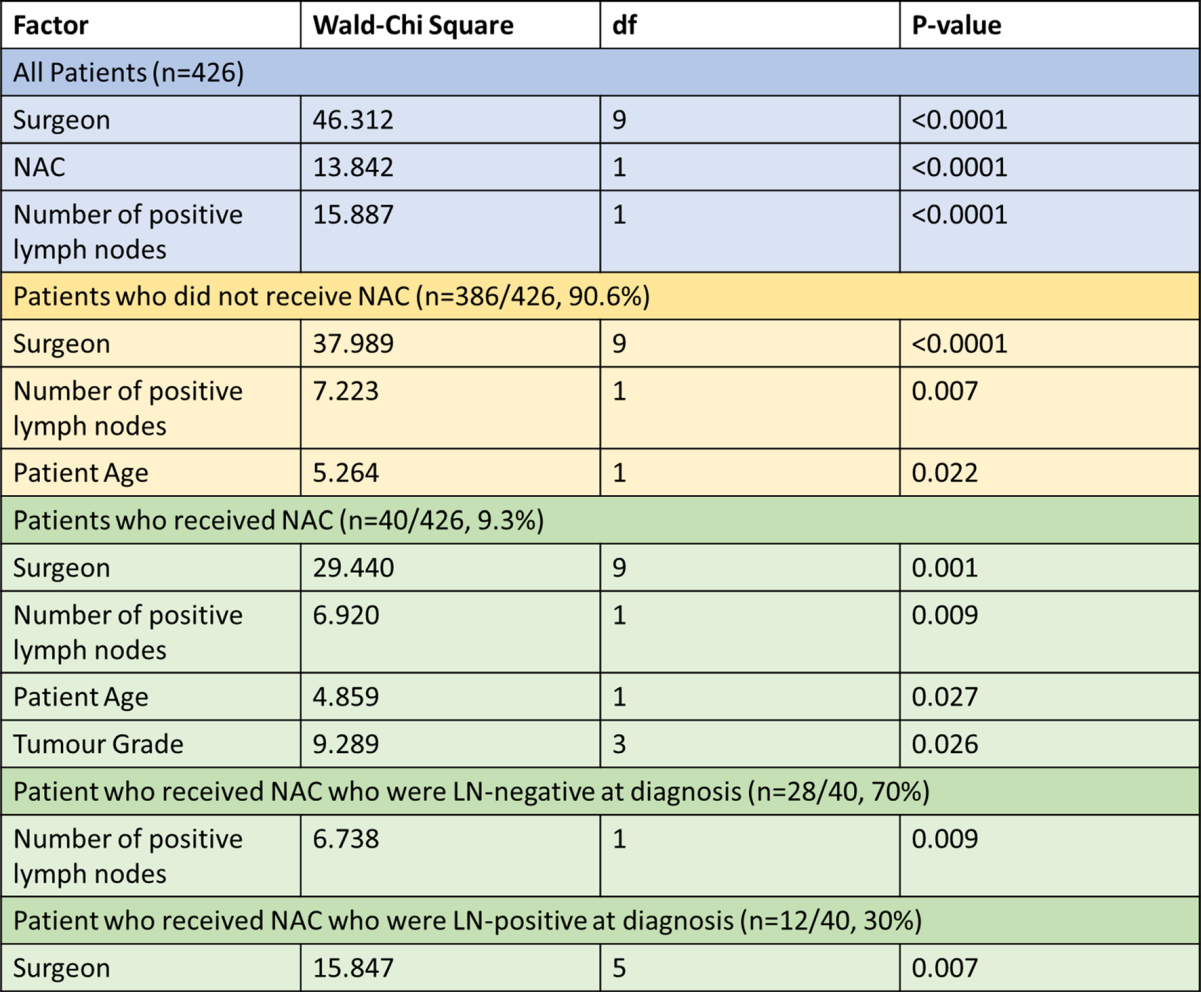
Significant explanatory variables from the multivariable generalised liner model analysis for the full cohort (blue) and subset patient cohorts (yellow & green)

Separate multivariable analyses were performed in the subsets of patients who did and did not receive NAC (*n* = 40 and *n* = 386 respectively). In both subsets the most statistically significant explanatory variables were surgeon, number of confirmed positive lymph nodes, and patient age, with higher numbers of sampled sentinel lymph nodes in younger patients who were subsequently found to have more extensive node positive disease. In the subset who received NAC, tumour grade was also found to be significant (Table 2).

Within the subset who received NAC (*n* = 40), separate multivariable analyses were performed for both the 30% of these patients who were lymph node positive at diagnosis and the 70% who were not. Only the number of confirmed positive sentinel lymph nodes correlated significantly with the number of SLNs biopsied in patients who were lymph node negative at diagnosis, with higher numbers of sentinel nodes removed in patients with node positive disease. The number of patients who were lymph node positive at diagnosis and received NAC was small (12 patients) but in this group, the surgeon was the only statistically significant explanatory variable correlating to the number of SLNs biopsied. Table 2 shows the results of the multivariate analysis for the subset patient cohorts. There was no significant association between the number of nodes removed and any subsequent axillary treatment.

## Discussion

This study has shown that a variety of factors have an impact on the number of sentinel nodes identified using isotope and blue dye that were removed during sentinel node biopsy. Some factors are easy to explain. Almost 10% of patients in this series had NAC. Significantly more SLN’s were removed in the group after NAC compared to patients who did not receive NAC. Almost two thirds of NAC patients had at least 3 sentinel nodes removed with an average of 3.5 nodes removed per patient. Recent guidance [7, 8] advocates that in patients who have proven lymph node metastases at diagnosis that revert to normal clinically post NAC then at least three nodes should be removed. In this study 83.3% of women in this group had three or more nodes removed, up to 40% of patients with involved nodes in the axilla can be converted to node negative by NAC [8]. Current optimal management includes marking at least one involved node at diagnosis and post NAC ensuring that the marked node together with two or more sentinel nodes are removed, a procedure known as targeted axillary dissection [4, 8, 9]. Our results are from a time when practice was evolving but our practice is consistent with guidelines and all patients in this series with involved nodes had a clip placed in one involved node and removal of the clipped node was achieved in all patients in this series.

Cancers that, develop in younger women are more likely to have a biologically aggressive phenotype [10] and are more likely to be triple negative or HER2 positive and be node positive. In this series younger women had significantly more sentinel nodes removed than older women. The same findings were seen with tumour size and tumour grade. Younger women, because they have larger tumours and higher grade tumours are more likely to be node positive. Surgeons know the patients age, tumour size and grade prior to surgery. Whether they consciously or unconsciously remove more nodes in such patients is not clear but that could explain why older patients in this series had less sentinel nodes removed. Alternatively, transmission of isotope and blue dye through lymphatics might reduce with age. Another explanation for these observations is that these factors are surrogates for node status explaining why these factors dropped out of significance in the multivariable analysis. Involved axillary nodes are palpable and thus are easier to identify and this is likely to explain why surgeons remove more sentinel nodes in patients whose nodes are involved.

Regardless of any influence of patient age or tumour biology, this study showed the most significant factor explaining the variation in the number of SLN’s removed was the surgeon. This demonstrates a potential weakness in the SLNB procedure because it is not completely standardised. Surgeon 2 removed an average of 3.3 nodes compared to surgeon 9 who took a mean of 1.8 (*p* < 0.0001) and surgeon 5 took a mean 1.9 (*p* < 0.001). Part of the variation can be explained by differences in the patients operated on by different surgeons. Some surgeons in this study had a higher percentage of patients who had sentinel node biopsies after NAC. For instance, Surgeon 10 operated on more patients who had NAC (28.6% of the operations they performed) and the mean size of tumours they operated on was 32.47 mm, compared to surgeon 4 who operated on the smallest proportions of patients who received NAC (4.3%) and the average size of tumours for this surgeon was 20.04 mm (Supplementary Fig. 3). Thus, some of the variation in the numbers of sentinel nodes removed between surgeons can be explained by other factors. Despite this, the multivariable model retained surgeon as a major factor. Whatever the explanation, there are significant differences between surgeons, and one must conclude that either some surgeons are taking too many nodes or others are taking too few.

Studies such as NSABP B-32 [5] showed that although there was a significant false negative rate with lower numbers of nodes removed, there was no obvious impact on the numbers of axillary recurrences. There is therefore no great concern that surgeons removing fewer axillary nodes will have a higher axillary recurrence rate. There is however evidence that because removing less nodes increases the false negative rate [4, 5]. Thus between 10 and 15% of patients having only one or two nodes have a false negative sentinel node and so may not receive appropriate systemic therapy given that axillary lymph node status plays an important role in adjuvant therapy decisions. Thus, removing less nodes results not only in qualitative information being lost (node positive or node negative), but also quantitative information on the numbers of nodes involved if less nodes are removed. Patients who have one out of one node involved may receive a different systemic treatment to a patient with two out of two or three out of three nodes involved so one can see how increasing the numbers of nodes retrieved at sentinel node biopsy might impact on adjuvant treatment decisions. We found no variation in axillary treatment related to the number of nodes removed.

In a large report based on SEER data [11], there was a significantly better survival for patients who had 3 nodes removed compared to patients with 1 or 2 involved nodes: Hazard Ratio of 0.73 CI 95% [0.60–0.88], *p* < 0.001. This improvement in survival was largely driven by the poorer outlook in the group who had only one SLN removed. The implications of this study are that the optimal number of sentinel lymph nodes that should be removed is three. The current study shows some surgeons consistently remove three or more nodes and others do not. This questions whether surgeons whose average lymph node retrieval rate is less than two are removing all the sentinel nodes and that this potentially under-stages some patients with subsequent implications for their systemic therapy and long-term outlook. What must also be considered is whether some surgeons are removing too many sentinel node. In Edinburgh, we have conducted a series of studies looking at the morbidity of both sentinel node biopsy as part of the UK ALMANAC study and axillary node sampling as part of a series of studies performed in Edinburgh [12–14]. Maxillary sampling involves removing four nodes from the lower maxilla by palpation and study of removing four nodes had minimal long-term arm morbidity in very carefully designed prospective studies [15]. Concerns about removing too many nodes are real but providing the number is four or less then our long-term data shows morbidity is very small in removing this number of maxillary lymph nodes. The current study suggests that the number of sentinel nodes removed per surgeon perhaps should become a quality measure collected by each surgeon and audited and considered at their annual appraisal. Surgeons removing consistently too many or too few sentinel nodes need then to amend their practice. Since the presentation of this study to the surgeons in the unit, there has been a clear change and the variation is now much less than it was during this study.

This study is strengthened by the sample size of 426 procedures from the largest breast unit in the UK and provides a meaningful analysis of current breast cancer care. A single centre analysis is important in removing some of the variables such as technique and consistent pathology reporting. The exclusions from the study were small because the Edinburgh Breast Unit collects all pathology data and pathology reporting is consistent in all cases. The largest amount of missing data was on grade and that was missing in only 3.99%. The study was largely successful at getting approximately 50 cases from each of the 10 surgeons. Most surgeons were consultants but also included were associate specialists and staff grade surgeons. All were experienced breast surgeons and no trainees were included in this study. Future analysis of this patient cohort to investigate the relationship between the number of SLN’s biopsied and adjuvant treatment as well as mortality would determine whether the number of nodes removed is a significant factor in relation to prognosis, however this was beyond the scope of this study at present.

## Electronic supplementary material

Below is the link to the electronic supplementary material.

Supplementary file1 (DOCX 256 kb)
