# Supplementary material for: Factors affecting the number of sentinel lymph nodes removed in patients having surgery for breast cancer
Source: Breast Cancer Res Treat. 2020 Aug 18;184(2):335–43. doi: 10.1007/s10549-020-05843-8 (PMC7599142; doi:10.1007/s10549-020-05843-8)
Supplement: Supplementary file 1 — Supplementary file1 (DOCX 256 kb) [file 10549_2020_5843_MOESM1_ESM.docx]

**Appendix**


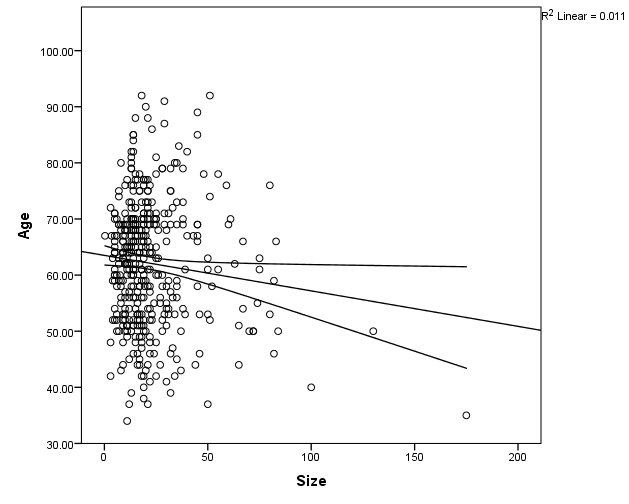


***Supplementary Figure 1:*** *Scatter plots of patient age versus tumour size. Lines represent linear fit with 95% confidence intervals.*


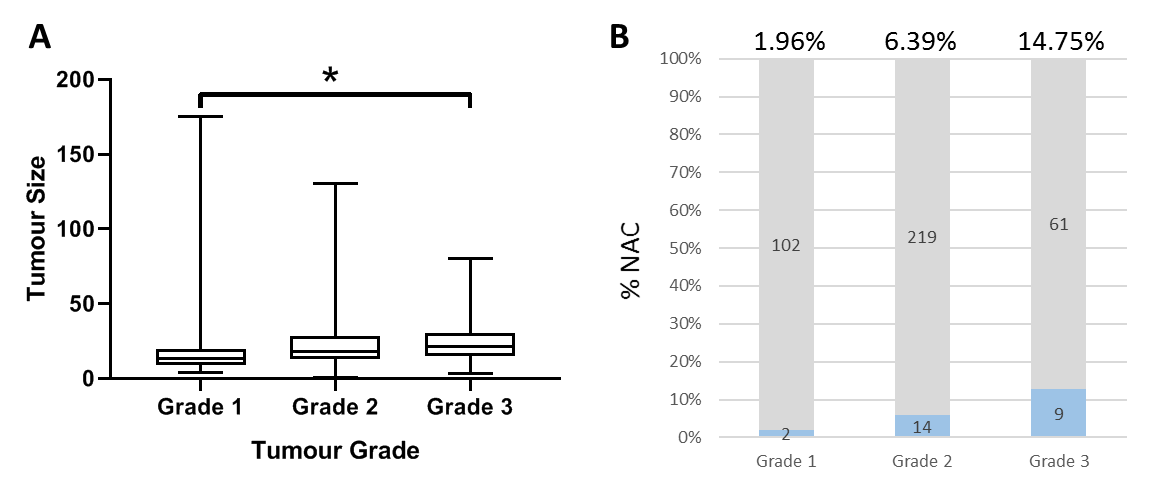


***Supplementary Figure 2:*** *(A) Boxplot showing distribution of tumour size by histological grade. Star represent p<0.05 based on ANOVA with Tukey’s test for multiple comparisons. (B) Proportional bar chart showing percentage of patients treated with NAC by histological grade.*


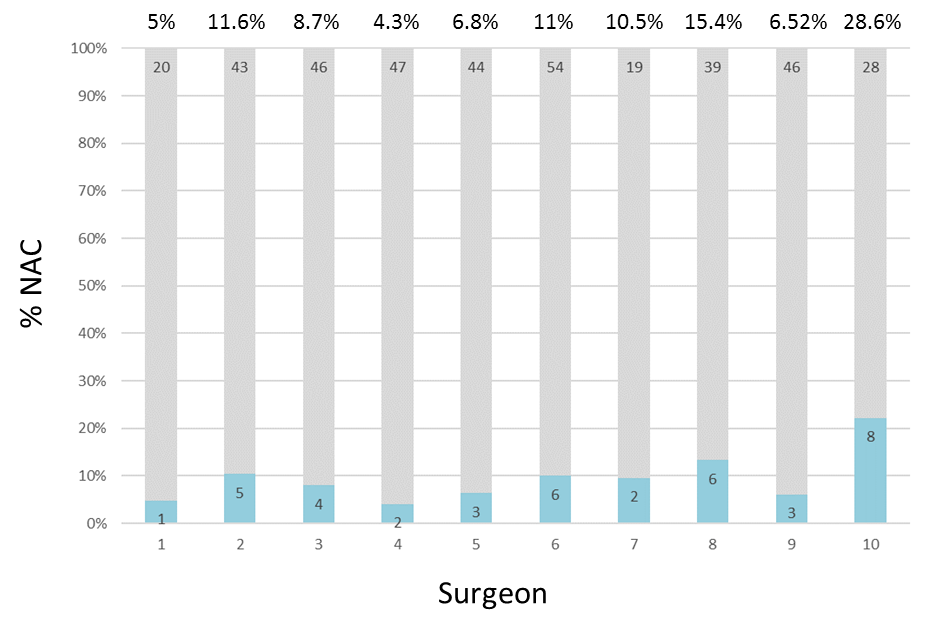


***Supplementary Figure 3:*** *Proportional bar chart showing percentage of patients treated with NAC by surgeon.*
